# The Lipid-Modulating Effect of Tangeretin on the Inhibition of Angiopoietin-like 3 (ANGPTL3) Gene Expression through Regulation of LXRα Activation in Hepatic Cells

**DOI:** 10.3390/ijms22189853

**Published:** 2021-09-12

**Authors:** Pei-Yi Chen, Tzu-Ya Chao, Hao-Jen Hsu, Chih-Yang Wang, Ching-Yen Lin, Wan-Yun Gao, Ming-Jiuan Wu, Jui-Hung Yen

**Affiliations:** 1 Center of Medical Genetics, Hualien Tzu Chi Hospital, Buddhist Tzu Chi Medical Foundation, Hualien 97004, Taiwan; pyc571@gmail.com; 2 Department of Molecular Biology and Human Genetics, Tzu Chi University, Hualien 97004, Taiwan; 109727103@gms.tcu.edu.tw (T.-Y.C.); jouyuan22@gmail.com (C.-Y.L.); 3 Department of Life Science, Tzu Chi University, Hualien 97004, Taiwan; hjhsu32@mail.tcu.edu.tw; 4 Program for Cancer Molecular Biology and Drug Discovery, Taipei Medical University, Taipei 11031, Taiwan; chihyang@tmu.edu.tw; 5 Graduate Institute of Cancer Biology and Drug Discovery, Taipei Medical University, Taipei 11031, Taiwan; 6 Institute of Medical Sciences, Tzu Chi University, Hualien 970, Taiwan; 102712131@gms.tcu.edu.tw; 7 Department of Biotechnology, Chia Nan University of Pharmacy and Science, Tainan 71710, Taiwan; imwu@gm.cnu.edu.tw

**Keywords:** TG-rich lipoproteins, tangeretin, lipoprotein lipase, ANGPTL3, LXRα

## Abstract

The excessive accumulation of TG-rich lipoproteins (TGRLs) in plasma is associated with dyslipidemia and atherosclerotic cardiovascular diseases (ASCVDs). Tangeretin is a bioactive pentamethoxyflavone mainly found in citrus peels, and it has been reported to protect against hyperlipidemia, diabetes, and obesity. The aim of this study was to investigate the lipid-modulating effects and the underlying mechanisms of tangeretin action in hepatic cells. Transcriptome and bioinformatics analyses with the Gene Ontology (GO) database showed that tangeretin significantly regulated a set of 13 differentially expressed genes (DEGs) associated with the regulation of lipoprotein lipase (LPL) activity. Among these DEGs, angiopoietin-like 3 (ANGPTL3), an essential inhibitor of LPL catalytic activity that regulates TGRL metabolism in plasma, was markedly downregulated by tangeretin. We demonstrated that tangeretin significantly inhibited the mRNA expression of ANGPTL3 in HepG2 and Huh-7 cells. Tangeretin treatment of hepatic cells also reduced the levels of both intracellular and secreted ANGPTL3 proteins. Moreover, we found that inhibition of ANGPTL3 production by tangeretin augmented LPL activity. We further demonstrated that the transcriptional activity of the ANGPTL3 promoter was significantly attenuated by tangeretin, and we identified a DNA element located between the −250 and −121 positions that responded to tangeretin. Furthermore, we found that tangeretin did not alter the levels of the nuclear liver X receptor α (LXRα) protein, an essential transcription factor that binds to the tangeretin-responsive element, but it can counteract LXRα-mediated ANGPTL3 transcription. On the basis of molecular docking analysis, tangeretin was predicted to bind to the ligand-binding domain of LXRα, which would result in suppression of LXRα activation. Our findings support the hypothesis that tangeretin exerts a lipid-lowering effect by modulating the LXRα-ANGPTL3-LPL pathway, and thus, it can be used as a potential phytochemical for the prevention or treatment of dyslipidemia.

## 1. Introduction

Patients with dyslipidemia, a major risk factor for atherosclerotic cardiovascular diseases (ASCVDs) and myocardial infarction (MI), often have high levels of total cholesterol, triglycerides (TGs), low-density lipoprotein cholesterol (LDL-C), and very low-density lipoprotein cholesterol (VLDL-C) and low levels of high-density lipoprotein cholesterol (HDL-C) in the blood circulation [1]. The excessive accumulation of TG-rich lipoproteins (TGRLs) in plasma is known to accompany dyslipidemia and ASCVDs [2,3]. TGRL particles, including chylomicrons and VLDLs, can be degraded by lipoprotein lipase (LPL) located on endothelial cells of capillary vessels, which may lead to decreased TG contents of lipoproteins and the release of free fatty acids in plasma. Released fatty acids can be absorbed and utilized by surrounding tissues such as the heart, muscle, and adipose tissue [4]. Recently, mounting evidence has shown that increased LPL activity can promote the clearance of plasma TG; however, LPL deficiency or loss of its enzymatic activity leads to TG accumulation in the plasma, causing hypertriglyceridemia [5]. Therefore, the modulation of LPL activity plays an essential role in regulating the amount of plasma TG and atherogenic lipoproteins and maintaining lipid homeostasis in circulation.

Recently, three members of the angiopoietin-like protein (ANGPTL) family that share structural similarity to vascular endothelial growth factors (angiopoietin proteins), including ANGPTL3, ANGPTL4 and ANGPTL8, have been shown to be important modulators of LPL catalytic activity and lipid metabolism [6,7,8,9,10,11]. Among these different ANGPTLs, ANGPTL3 is known to be exclusively produced in the liver with functions similar to those of a hepatokine and plays the most essential role in the modulation of TGRL metabolism [12]. The *ANGPTL3* gene is located on chromosome 1p31.1, and it encodes a 460-amino acid glycoprotein. The mature protein secreted by hepatocytes has a molecular weight of approximately 70 kDa [13]. The protein structure of ANGPTL3 contains several regions, an N-terminal coiled-coil domain, a linker region, and a C-terminal fibrinogen-like domain [14,15]. The N-terminal domain acts as a potent inhibitor of LPL and endothelial lipase (EL) activity [16,17]. ANGPTL3 is mostly activated after feeding and cooperates with ANGPTL8 to reduce the levels of plasma TG through reversible inhibition of LPL activity [18,19]. In ANGPTL3-defective or *Angptl3*^−/−^ mice with various genetic backgrounds, plasma TG and cholesterol levels are markedly reduced [8]. In dyslipidemic mice, suppression of ANGPTL3 activity by a monoclonal antibody evinacumab effectively decreases plasma TG and cholesterol levels and markedly reduces atherosclerotic lesions [20]. In humans, loss-of-function variants of *ANGPTL3* are associated with lower levels of TG, LDL-C, and HDL-C in the blood circulation [20,21,22]. Additionally, ANGPTL3 plasma levels may be associated with arterial wall thickness and could serve as an early predictor of peripheral arterial diseases [23]. In human clinical trials, evinacumab administration resulted in a reduction in fasting TG and LDL-C levels [24,25]. Similar lipid-lowering effects were achieved by the use of antisense oligonucleotides (ASOs) targeting hepatic ANGPTL3 mRNA [26]. Thus, pharmacological inhibition of ANGPTL3 activity or downregulation of its gene expression is a promising approach to modulating plasma TGRLs and reducing the risk of ASCVDs [27,28].

Several antioxidant phytochemicals, such as flavonoids, exert anti-inflammatory and lipid-modulating activities. The development of ANGPTL3 inhibitors from natural antioxidant flavonoids may lead to effective strategies against dyslipidemia. Tangeretin (4′,5,6,7,8-pentamethoxyflavone) is a dietary bioactive polymethoxyflavone (PMF) that is found in citrus peel [29]. Tangeretin has been reported to possess several beneficial antioxidant, anti-inflammatory, anticancer, antilipogenetic, antidiabetic, and anti-obesity activities [30,31,32,33,34]. Recently, in vitro and in vivo studies have focused on the effects of tangeretin on dyslipidemia. Tangeretin reduced the intracellular synthesis of TG, free cholesterol, and cholesterol esters and regulated apolipoprotein-B-containing lipoprotein secretion in the HepG2 human cell line, suggesting that it has potential effects against hyperlipidemia [35]. Citrus peel extracts with abundant PMFs exhibited hypolipidemic effects in diet-induced hypercholesterolemic hamsters, and these effects were associated with extensive absorption of tangeretin in vivo [36]. Zeng et al. reported that citrus PMFs containing a high level of tangeretin downregulated the mTOR-P70S6K-SREBP pathway in hepatic cells and attenuated metabolic syndrome by modulating the gut microbiota in high-fat-diet-fed mice [37]. Feng et al. reported that tangeretin could ameliorate obesity, dyslipidemia, and hepatic steatosis by modulating the expression of genes involved in lipogenesis and lipid oxidation in high-fat-diet-fed rats [38]. Tangeretin has been shown to exert lipid-lowering effects and improve dyslipidemia in vitro and in vivo, but the underlying mechanisms remain unclear. In this study, we aimed to investigate the antidyslipidemic effects of tangeretin with special attention to the regulation of ANGPTL3 expression and the underlying molecular mechanisms of its action in hepatic cells.

## 2. Results

### 2.1. Effects of Tangeretin on the Viability of Hepatic Cell Lines

**Figure 1:**
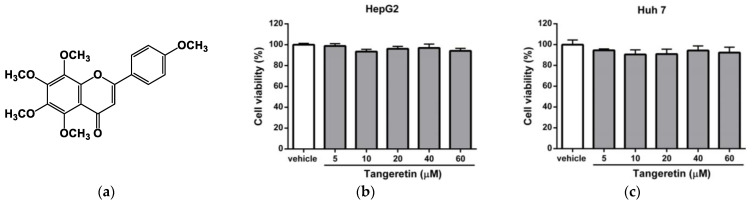
The effect of tangeretin on HepG2 and Huh-7 cell viability. (**a**) Chemical structure of tangeretin (4′,5,6,7,8-pentamethoxyflavone). (**b**) HepG2 and (**c**) Huh-7 cells were treated with vehicle (0.1% DMSO) or tangeretin (5, 10, 20, 40, and 60 μM) for 24 h. The viability of the cells was measured by MTT assay. The data are presented as the means ± SD of three independent experiments.

Before the examination of molecule’s regulating effect, we need to be sure that it does not have detrimental effects in tested cells; thus, we examine the cytotoxic effect of tangeretin (Figure 1a) on hepatic cells. HepG2 and Huh-7 cells were incubated with the vehicle (0.1% DMSO) or tangeretin (5, 10, 20, 40, and 60 μM) for 24 h, and the viability of the cells was measured by MTT assay. As shown in Figure 1b,c, compared with the vehicle, tangeretin (5–60 μM) induced no significant cytotoxicity in HepG2 and Huh-7 cells.

### 2.2. Analysis of Tangeretin-Regulated Gene Expression and Biological Processes in Hepatic Cells

**Figure 2:**
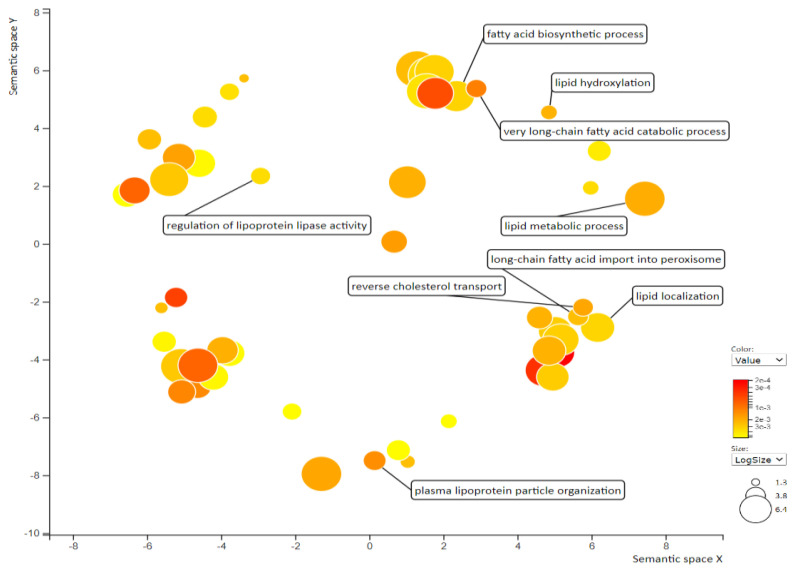
Enrichment analysis and visualization of Gene Ontology (GO) by REVIGO. Significantly enriched GO biological process (BP) in tangeretin (40 μM)-treated HepG2 cells was analyzed by REVIGO. The scatterplot indicates functional clusters; the bubble color and size represent the *p*-values and the frequency of the GO term in the underlying database, respectively.

Transcriptome analysis is a valuable method to elucidate molecular changes in response to chemical agents for making decisions in the early stage of drug discovery [39]. Thus, to investigate the potential genes involved in the tangeretin-mediated regulation of TGRLs and dyslipidemia, we examined the differentially expressed genes (DEGs) in tangeretin-treated hepatic cells by human genome-wide microarray analysis. The entire set of significant DEGs (*p* < 0.05) in tangeretin-treated cells was subjected to functional annotation with Gene Ontology (GO) term enrichment analysis to determine the biological processes (BPs) in which they are involved. Supplementary Table S1 shows the 50 GO BP terms with the most significant *p* values. Furthermore, REVIGO was performed to summarize the long lists of GO terms on the basis of semantic similarities to visualize nonredundant GO annotations for interpretation. It was found that lipid modulation-related BPs, such as plasma lipoprotein particle organization, reverse cholesterol transport, lipid metabolic process, lipid hydroxylation, fatty acid biosynthetic process, lipid localization, and regulation of lipoprotein lipase activity, were significantly changed in the tangeretin-treated cells (Figure 2).

**Figure 3:**
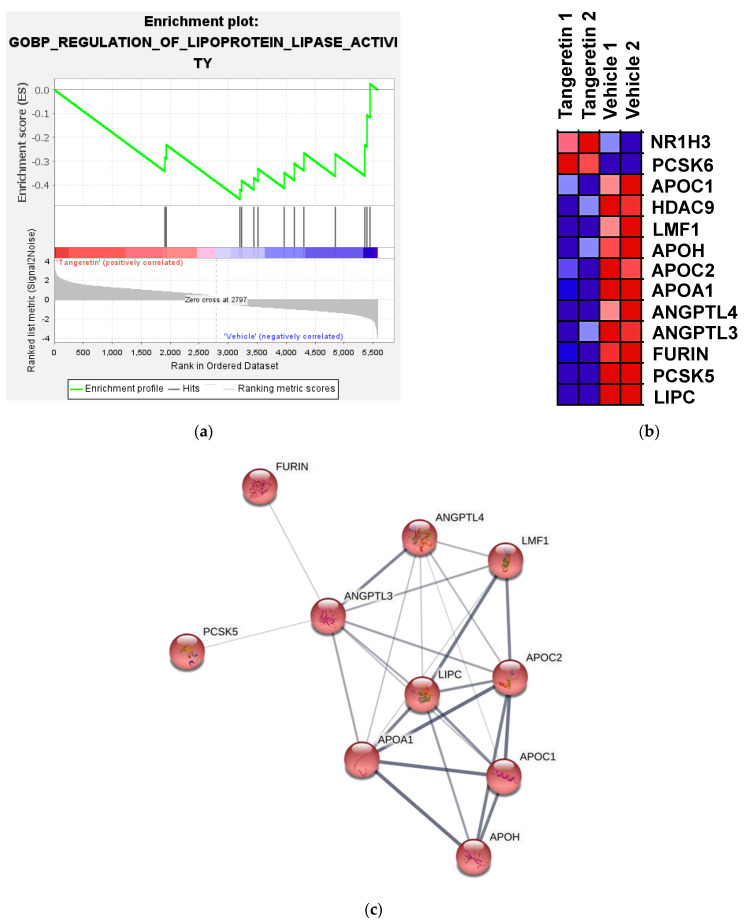
Pathway enrichment, GSEA, and prediction of protein–protein interactions for DEGs in response to tangeretin treatment. The differentially expressed genes (DEGs) in tangeretin-treated HepG2 cells were analyzed by a human genome-wide microarray as described in the Materials and Methods. (**a**) Gene set enrichment analysis (GSEA) demonstrates that the GO BP signature “Enrichment plot: GO BP Regulation of Lipoprotein Lipase Activity” (GO:0051004) gene set is enriched with the DEGs of tangeretin-treated cells. The barcode plot represents the position of the genes in the gene set. The horizontal bar in graded color from red to blue indicates positive and negative regulation by tangeretin. The vertical axis in the lower plot indicates the ranked list metric. (**b**) The DEGs in the gene set of GO:0051004 in response to tangeretin (40 μM) treatment. (**c**) Predicted protein–protein interaction for tangeretin-downregulated genes associated with regulation of LPL activity. The corresponding genes were uploaded to the STRING interaction database. Disconnected nodes were omitted, and line thickness indicates the strength of the data supporting the connections.

The regulation of lipoprotein lipase (LPL) activity is important for the modulation of plasma TGRLs and TGs homeostasis. Therefore, we focused on the GO BP of regulation of lipoprotein lipase activity (GO:0051004) and identified consistent gene expression changes involved in this BP by performing gene set enrichment analysis (GSEA) [40]. A search for gene sets that were significantly enriched at a false discovery rate (FDR) < 0.25 for tangeretin vs. vehicle was carried out. As shown in Figure 3a,b, an enrichment plot showed that a gene set with 13 DEGs associated with GO:0051004 was significantly regulated by tangeretin treatment. Among these 13 DEGs, 11 gene transcripts, namely *LIPC*, *PCSK5*, *FURIN*, *ANGPTL3*, *ANGPTL4*, *APOA1*, *APOC2*, *APOH*, *LMF1*, *HDAC9*, and *APOC1,* were significantly downregulated in the tangeretin-treated cells, as shown in Table 1. We then uploaded these 11 genes into the STRING interaction database for the prediction of protein–protein interactions (PPIs). The Markov clustering algorithm (MCL) (inflation factor = 3) was applied to reveal the cluster structure in graphs [41]. As shown in Figure 3c, 10 proteins in one cluster in the PPI network were shown. Among these proteins, ANGPTL3 has eight edges within the cluster and is considered a major hub. ANGPTL3 has been reported to be mainly involved in the suppression of LPL activity to affect atherogenic TGRL metabolism and dyslipidemia [42]. Thus, ANGPTL3 was chosen for further investigation.

### 2.3. Effects of Tangeretin on ANGPTL3 mRNA and Protein Expression in Hepatic Cells

**Figure 4:**
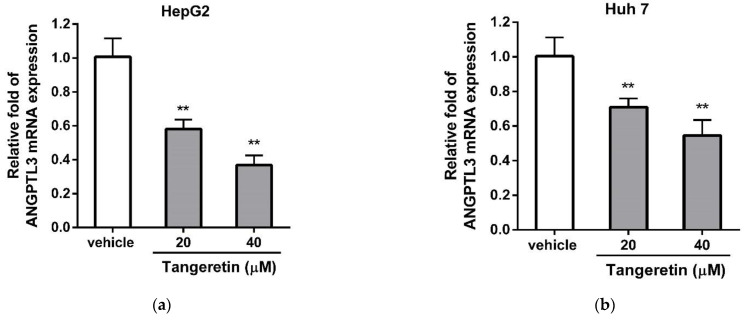

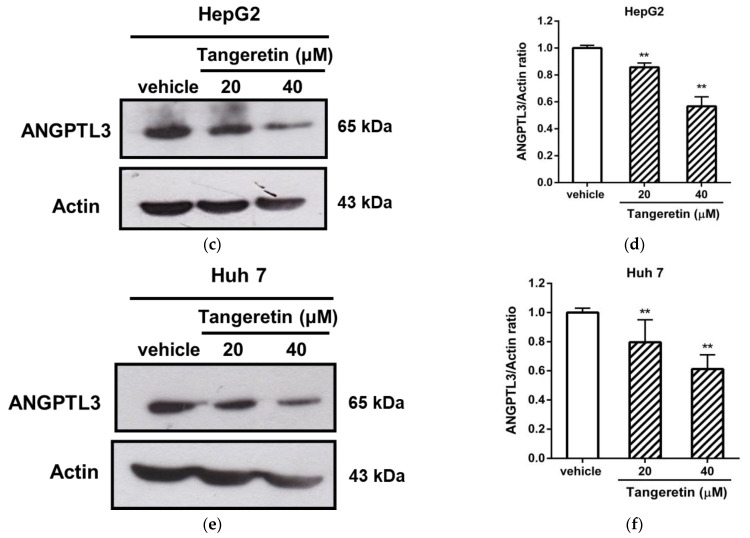
The effect of tangeretin on ANGPTL3 expression in HepG2 and Huh-7 cells. HepG2 and Huh-7 cells were treated with vehicle (0.1% DMSO) or tangeretin (20 and 40 μM) for 24 h. The mRNA expression of ANGPTL3 in (**a**) HepG2 and (**b**) Huh-7 cells was measured by RT-Q-PCR analysis. The data represent the mean ± SD of three independent experiments. The ANGPTL3 proteins in HepG2 and Huh-7 cells were determined by Western blot analysis. (**c**,**e**) Representative blots are shown. (**d**,**f**) The normalized intensity of ANGPTL3 versus actin protein is presented as the mean ± SD of three independent experiments. ** *p* < 0.01 indicates significant differences compared to the vehicle-treated cells.

To investigate whether tangeretin downregulated the gene expression of ANGPTL3 in hepatic cells, we validated the effect of tangeretin on ANGPTL3 mRNA expression in HepG2 and Huh-7 cells using RT-Q-PCR analysis. As shown in Figure 4a, tangeretin (20 and 40 μM) significantly suppressed the mRNA expression of ANGPTL3 by 0.58 ± 0.06-fold and 0.37 ± 0.06-fold in HepG2 cells, respectively, compared to the expression in the vehicle-treated cells (1.00 ± 0.11) (*p* < 0.01). Similar results also showed that tangeretin (20 and 40 μM) reduced the mRNA level of ANGPTL3 in Huh-7 cells by 0.71 ± 0.05-fold and 0.55 ± 0.09-fold, respectively, compared with the vehicle-treated group (1.00 ± 0.10) (*p* < 0.01) (Figure 4b). Furthermore, we examined the effect of tangeretin on ANGPTL3 protein expression in hepatic cells using Western blot analysis. As shown in Figure 4c,d, HepG2 cells treated with tangeretin (20 and 40 μM) exhibited significantly reduced protein levels of ANGPTL3 (0.84 ± 0.02-fold and 0.60 ± 0.03-fold, respectively) compared to vehicle-treated HepG2 cells (1.00 ± 0.02) (*p* < 0.01). A similar inhibitory effect of tangeretin on ANGPTL3 protein expression in Huh-7 cells is shown in Figure 4e,f. These data indicate that tangeretin inhibited the mRNA and protein expression of ANGPTL3 in hepatic cells.

### 2.4. A Tangeretin-Mediated Reduction in ANGPTL3 Restores Lipoprotein Lipase (LPL) Activity in Hepatic Cells

**Figure 5:**
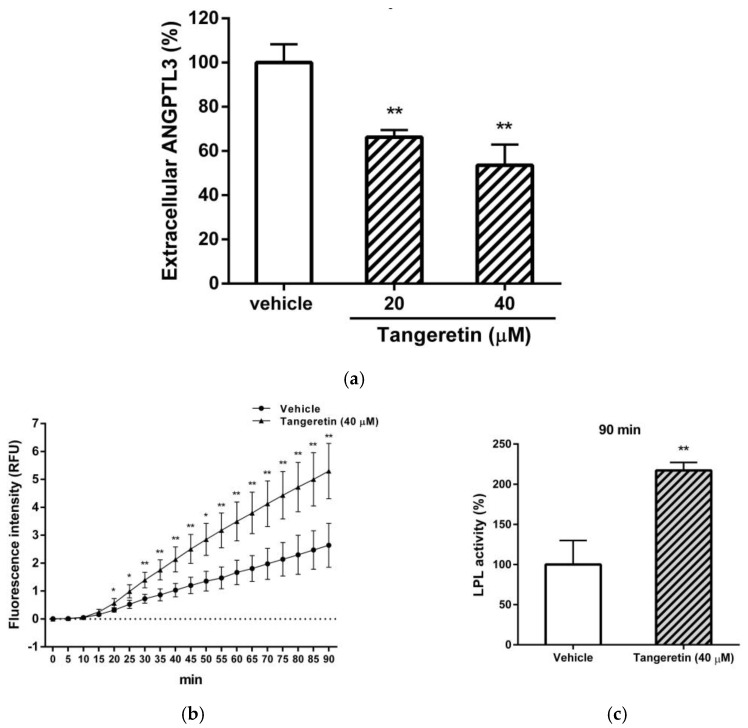
The effect of the tangeretin-mediated reduction of ANGPTL3 on LPL activity. HepG2 cells were treated with vehicle (0.1% DMSO) or tangeretin (20 and 40 μM) for 24 h. (**a**) The culture medium was collected, and the extracellular ANGPTL3 proteins were determined by ELISA as described in the Materials and Methods. (**b**) HepG2 cells were treated with vehicle or tangeretin (40 μM) for 24 h, and extracellular protein samples were prepared as described in the Materials and Methods. The protein samples (50 μg) and LPL proteins were incubated with substrates to determine the catalytic activity of the LPL enzyme in a fluorogenic analysis. The fluorescence intensity was measured for 90 min, and the data represent the mean ± SD of three independent experiments. * *p* < 0.05 and ** *p* < 0.01 represent significant differences compared to the vehicle-treated group. (**c**) Fluorescence intensity (LPL activity) at t = 90 min relative to that of the vehicle-treated group. The data represent the mean ± SD from three independent experiments. ** *p* < 0.01 represents significant differences compared to the vehicle-treated group.

The amounts of secreted ANGPTL3 proteins in the tangeretin-treated cells were analyzed by ELISA. As shown in Figure 5a, tangeretin (20 and 40 μM) significantly reduced the levels of ANGPTL3 protein secreted into the medium by approximately 34% and 47%, respectively. Moreover, we also investigated whether the tangeretin-mediated reduction in ANGPTL3 can restore LPL catalytic activity. As shown in Figure 5b, the LPL activity was significantly increased in a time-dependent manner by extracellular proteins collected from tangeretin-treated cell culture supernatants compared to that of the culture medium collected from vehicle-treated cells. After LPL incubation for 90 min, extracellular proteins of tangeretin-treated cells elevated the LPL catalytic activity up to 217.17 ± 9.90% compared with those of the vehicle-treated cells (100.00 ± 29.86%) (Figure 5c). These data indicate that the reduction in secreted ANGPTL3 protein induced by tangeretin led to the augmentation of LPL activity.

### 2.5. Tangeretin Inhibits the Transcriptional Activity of the ANGPTL3 Promoter in Hepatic Cells

**Figure 6:**
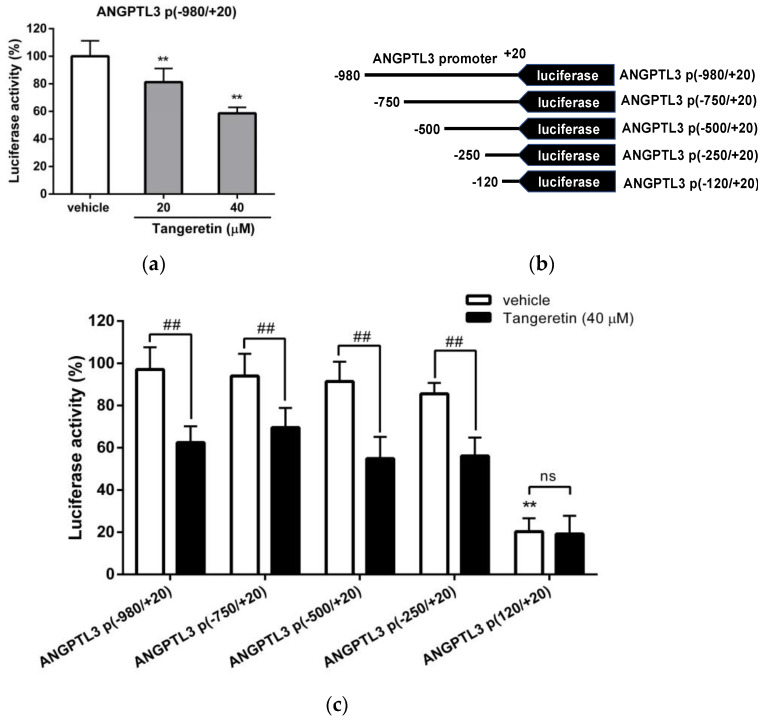
The effect of tangeretin on ANGPTL3 promoter activity. (**a**) HepG2 cells were cotransfected with an ANGPTL3 promoter–reporter plasmid (ANGPTL3 p(−980/+20)) and a *Renilla* control plasmid for 24 h. The cells were then treated with vehicle (0.1% DMSO) or tangeretin (20 and 40 μM) for 24 h. The luciferase activities were measured and normalized to their respective *Renilla* luciferase activities. The data represent the mean ± SD from three independent experiments. ** *p* < 0.01 indicates significant differences compared to the vehicle-treated cells. (**b**) Reporter plasmids containing serially deleted DNA fragments of the ANGPTL3 promoter. (**c**) HepG2 cells were cotransfected with serially deleted ANGPTL3 promoter–reporter plasmids and a *Renilla* plasmid for 24 h and then treated with vehicle or tangeretin (40 μM) for an additional 24 h. The luciferase activities were measured and normalized to the respective *Renilla* luciferase activity. The data represent the mean ± SD of three independent experiments. ** *p* < 0.01 indicates a significant difference compared to vehicle-treated ANGPTL3 p(−980/+20) plasmid-transfected cells. ## *p* < 0.01 indicates significant differences compared to the respective vehicle-treated cells. “ns” indicates no significance.

The aforementioned data suggest that tangeretin can inhibit ANGPTL3 expression at the transcriptional level; thus, we further investigated the effect of tangeretin on the transcriptional activity of the *ANGPTL3* promoter in hepatic cells. As shown in Figure 6a, tangeretin (20 and 40 μM) decreased the luciferase activity to 81.27 ± 9.92% and 58.70 ± 4.25%, respectively, compared to that of vehicle-treated cells (100.00 ± 11.21) (*p* < 0.01). These data indicate that tangeretin can suppress the transcriptional activity of the *ANGPTL3* promoter in hepatic cells.

To further investigate the mechanism underlying the tangeretin-mediated downregulation of ANGPTL3 expression, the regulatory elements of the *ANGPTL3* promoter responsive to tangeretin were identified. The deletion constructs of *ANGPTL3* promoter including ANGPTL3 p(−750/+20), ANGPTL3 p(−500/+20), ANGPTL3 p(−250/+20), and ANGPTL3 p(−120/+20) (Figure 6b), and the *Renilla* control were cotransfected into HepG2 cells, followed by incubation with vehicle or tangeretin (40 μM). As shown in Figure 6c, in HepG2 cells transfected with ANGPTL3 p(−750/+20), ANGPTL3 p(−500/+20) or ANGPTL3 p(−250/+20), tangeretin significantly decreased luciferase activity compared to that measured in vehicle-treated cells. However, the transcriptional activity was markedly decreased and showed no response to tangeretin treatment in cells transfected with the ANGPTL3 p(−120/+20) plasmid. These results indicate that the ANGPTL3 promoter between the −250 and −121 positions is the essential DNA region for ANGPTL3 transcription and contains a responsive element for the tangeretin-mediated inhibition of ANGPTL3 gene expression.

### 2.6. Effects of Tangeretin on LXRα-Mediated ANGPTL3 Expression in Hepatic Cells

**Figure 7:**
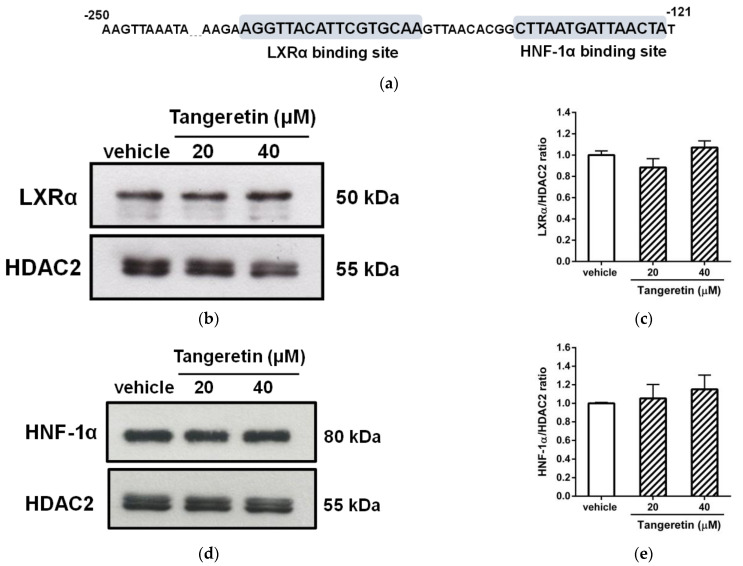
Effects of tangeretin on nuclear LXRα and HNF-1α proteins. (**a**) Analysis of the tangeretin-responsive element within the ANGPTL3 promoter (−250/−121). The DNA sequences identified by the binding of the transcription factors LXRα and HNF-1α are indicated as the LXRα-binding site and HNF-1α-binding site, respectively. (**b**) HepG2 cells were treated with vehicle (0.1% DMSO) or tangeretin (20 and 40 μM) for 24 h. The level of nuclear LXRα protein was analyzed by Western blot analysis. A representative blot is shown. (**c**) The normalized intensity of LXRα versus HDAC2 is presented as the mean ± SD of three independent experiments. (**d**) The level of nuclear HNF-1α protein was analyzed by Western blot analysis. A representative blot is shown. (**e**) The normalized intensity of HNF-1α versus HDAC2 is presented as the mean ± SD of three independent experiments.

As shown in Figure 7a, the sequence between nucleotides −250 and −121 within the *ANGPTL3* promoter in response to tangeretin was analyzed. Within this DNA region, regulatory elements for the interaction of two putative transcription factors, LXRα (−163 to −147) and HNF-1α (−136 to −122), have been reported to be involved in the regulation of ANGPTL3 gene expression in hepatocytes [43,44]. To determine whether these two transcription factors are involved in the tangeretin-mediated downregulation of ANGPTL3, the effect of tangeretin on the levels of nuclear LXRα and HNF-1α proteins was determined. As shown in Figure 7b–e, when cells were treated with tangeretin, the amounts of nuclear LXRα and HNF-1α proteins were not significantly changed in HepG2 cells.

**Figure 8:**
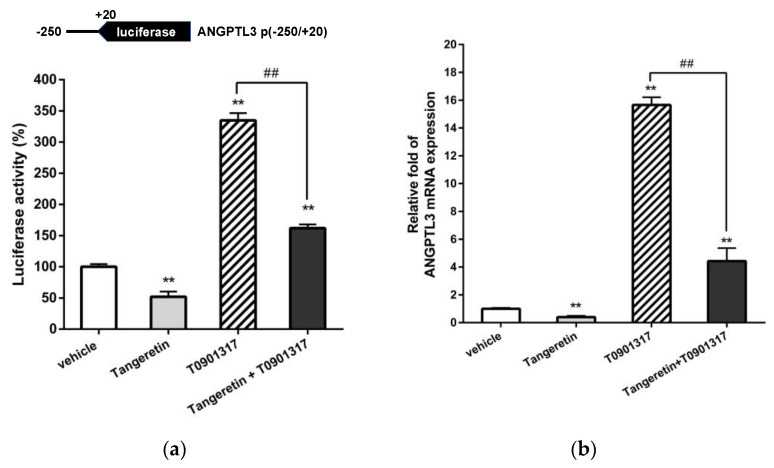
Effects of tangeretin on ANGPTL3 transcription upon T0901317-induced LXRα activation in HepG2 cells. (**a**) HepG2 cells were cotransfected with an ANGPTL3 promoter–reporter plasmid (ANGPTL3 p(−250/+20)) and a *Renilla* control plasmid for 24 h. These plasmid-transfected cells were pretreated with vehicle or tangeretin (40 μM) for 1 h followed by treatment with T0901317 (1 μM) for an additional 24 h. The luciferase activities were measured and normalized to their respective *Renilla* luciferase activities. The data represent the mean ± SD from three independent experiments. (**b**) HepG2 cells were pretreated with vehicle or tangeretin (40 μM) for 1 h followed by treatment with T0901317 (1 μM) for an additional 24 h. The mRNA levels of ANGPTL3 were measured by RT-Q-PCR analysis. ** *p* < 0.01 indicates a significant difference compared to vehicle-treated cells. ## *p* < 0.01 indicates significant differences compared to the T0901317 alone-treated cells.

ANGPTL3 expression has been reported to be increased through LXRα-dependent transcriptional activation in hepatic cells. Thus, the ANGPTL3 p(−250/+20) constructs were transfected into HepG2 cells to examine the effect of tangeretin on LXRα-induced ANGPTL3 transcription. As shown in Figure 8a, treatment with T0901317 alone significantly increased ANGPTL3 promoter activity. The T0901317-induced transcriptional activity was significantly suppressed by cotreatment with tangeretin. Moreover, the level of ANGPTL3 mRNA elevated by T0901317 could also be significantly attenuated by tangeretin treatment in HepG2 cells (Figure 8b). These data reveal that LXRα-mediated ANGPTL3 mRNA transcription can be significantly inhibited by tangeretin. Our findings suggest that tangeretin downregulates ANGPTL3 expression through modulation of LXRα activity in hepatic cells.

### 2.7. Tangeretin Docks to the Ligand-Binding Domain of the LXRα Protein

**Figure 9:**
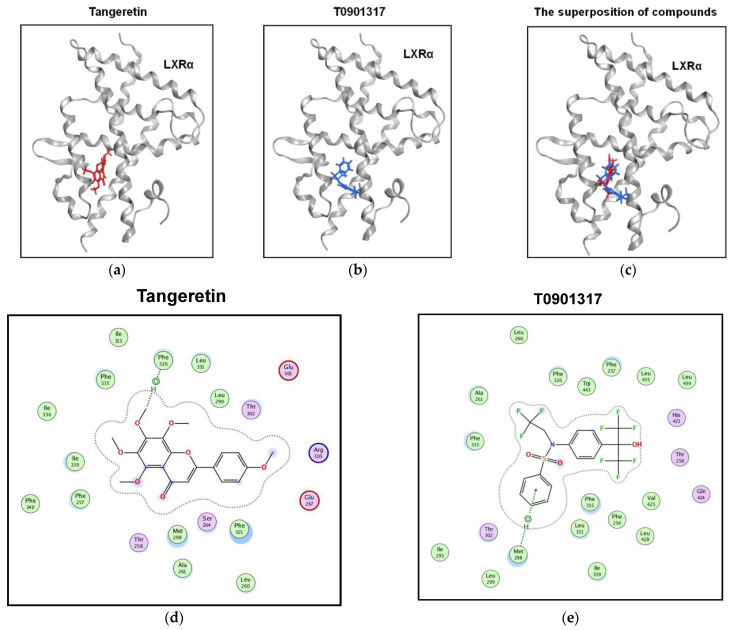
Tangeretin can dock into the ligand-binding domain of LXRα. The poses of tangeretin and T0901317 docked preferentially to the ligand-binding domain of LXRα. LXRα is shown as a gray ribbon, and the compounds are shown in (**a**) red (tangeretin) and (**b**) blue (T0901317). (**c**) The superposition of both tangeretin and T0901317 docked to the ligand-binding pocket of LXRα. Two-dimensional interaction map of LXRα with (**d**) tangeretin and (**e**) T0901317. The hydrophobic and hydrophilic amino acid residues near the compounds are colored green and pink, respectively.

To investigate whether tangeretin possesses the potential to interact with the LXRα protein, a molecular docking program to predict the protein binding ability of tangeretin was used as described in the Materials and Methods. As shown in Figure 9a,b, the docking results show that both tangeretin and T0901317 can bind to LXRα to form similar docking poses with docking scores of −8.64 and −8.71, respectively, which may indicate that these two compounds possess similar binding affinities for LXRα. The superposition data showed that tangeretin and T0901317 specifically docked into the ligand-binding domain (LBD) of LXRα (Figure 9c). The ligand–receptor interaction maps showed that there are several LXRα hydrophobic amino acid residues (L299, L331, F326, F335, I336, I339, F257, M298, and F315) near tangeretin (Figure 9d). The interaction maps were also found for T0901317 bound to the LXRα receptor, and the results showed that T0901317 is surrounded by hydrophobic residues (Figure 9e), which was consistent with our previous studies [45]. These data indicate that both tangeretin and the T0901317 ligand can bind to similar hydrophobic pockets in the LBD of LXRα.

## 3. Discussion

**Figure 10:**
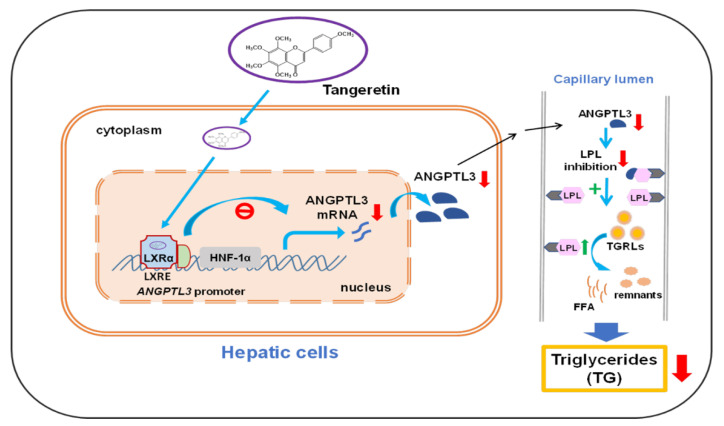
A hypothetical model for the regulation of the LXRα-ANGPTL3-LPL pathway by tangeretin. Tangeretin inhibits the mRNA and protein expression of ANGPTL3 by counteracting LXRα-mediated transcriptional activation in hepatic cells, resulting in a restoration of LPL activity, increases in TGRL metabolism, and a reduction in TG levels in circulation.

The lipid-lowering effects of citrus tangeretin on the regulation of dyslipidemia have been reported in numerous studies. To our knowledge, this is the first report to show that tangeretin inhibits the gene expression of hepatic ANGPTL3 via counteracting the activity of LXRα, a key regulator for lipid homeostasis. The decreases in intracellular and secreted ANGPTL3 proteins by tangeretin may augment LPL catalytic activity during the hydrolysis of TGs in lipoproteins, resulting in a reduction of TG accumulation in circulation (Figure 10).

Tangeretin is one of the most abundant PMFs found in citrus peels and has been shown to protect against dyslipidemia, obesity, hepatic steatosis, and atherosclerosis. Oral administration of tangeretin in mice did not impair organ function, which indicates that tangeretin is a nontoxic agent and can be safe to use in vivo [46]. In this study, tangeretin (5–60 μM) induced no significant cytotoxic effects in hepatic cells. These results support the supposition that tangeretin is a safe flavonoid phytochemical and can serve as a functional ingredient in food. However, similar to several other flavonoids, tangeretin exhibits limited solubility and absorption in the gastrointestinal tract and poor oral bioavailability, which may make it less effective in vivo [47]. Manthey et al. reported that oral administration of tangeretin (50mg kg^−1^ of body weight) by gavage in rats, its aglycone metabolite exhibited the highest concentrations between 4 and 6 h (~0.38 μg/mL). The glucuronide metabolites of tangeretin showed a broad t_max_ range between 3 and 6 h with the highest concentration of 1.45 μg/mL [47]. Hung et al. reported on the pharmacokinetics and bioavailability of orally administered tangeretin (50 mg kg^−1^ of bodyweight) in SD rats and found that the maximal concentrations (C_max_) of tangeretin in plasma was 0.87 ± 0.33 μg/mL (equivalent to 2.34± 0.09 μM), the time to reach the maximum concentration (t_max_) was 340.00 ± 48.99 min, and the half-life (t_1/2_) was 342.43 ± 71.27 min [48]. In this study, we demonstrated that tangeretin in hepatic cells was effective at 20 and 40 μM. These concentrations were used to treat several cells lines, although they are higher than the concentration that can be used in vivo. To improve the oral bioavailability of tangeretin in vivo, novel encapsulated compounds or formulations employed in emulsion-based delivery systems may enhance its solubility and stability to achieve the effective concentrations that were used in this in vitro cell study [49,50].

In this study, transcriptome analysis demonstrated the molecular mechanisms that respond to tangeretin to modulate lipid homeostasis. Using pathway enrichment and REVIGO analyses of microarray data, tangeretin was found to modulate the expression of a gene set involved in the regulation of LPL activity. Performing GSEA, 11 genes associated with the regulation of LPL activity were downregulated in tangeretin-treated cells. Among these genes, the mRNA levels of four DEGs, hepatic lipase (*LIPC*), proprotein convertase subtilisin/kexin type 5 (*PCSK5*), proprotein convertase subtilisin/kexin type 3 (*FURIN/PCSK3)*, and *ANGPTL3* were the most reduced (~ ≤ 0.5-fold) by tangeretin. Hepatic lipase (HL) is an enzyme that can hydrolyze the TG and phospholipid portions to convert IDL to LDL, produce small and dense LDL molecules, and remodel HDL particles in plasma [51]. The role of HL as a pro- or anti-atherogenic enzyme in humans remains unclear. In animal and human studies, HL deficiency has been reported to be associated with premature coronary artery disease [52,53]. In contrast to these studies, another study reported that HL deficiency increased cholesterol levels but reduced the risk of atherosclerosis [54]. The determination of whether tangeretin downregulates LIPC expression or is involved in antiatherogenic processes requires further clarification. It has been found that PCSK5 and furin/PCSK3 can cleave and inactivate the catalytic activity of EL and LPL, which play essential roles in chylomicron, VLDL, and HDL metabolism. In addition to EL and LPL, the full-length ANGPTL3 protein was found to possess amino acid residues in the linker region for furin cleavage [55]. This N-terminal truncation of ANGPTL3 by furin cleavage was reported to promote the inhibitory effect on EL but not LPL, suggesting that the furin-mediated ANGPTL3 effect is more critical for the inhibition of EL activity [56]. Moreover, furin and PCSK5 can enhance the inflammatory response in atherosclerosis [57]. Furin is mainly expressed in the liver and is the most dysregulated protein convertase in human atherosclerotic arteries. Drugs that inhibit furin in arteries may modulate the development of ASCVDs [58]. In this study, both hepatic PCSK5 and furin mRNA expression were downregulated by tangeretin. Whether tangeretin has the potential to modulate EL or LPL activity via alleviation of hepatic PCSK5 and furin levels requires further investigation.

In this study, we found that tangeretin decreased the mRNA and protein expression of ANGPTL3 in hepatic cells. The secreted form of the ANGPTL3 protein was also significantly reduced in tangeretin-treated cells. Our findings indicate that tangeretin can markedly attenuate hepatic ANGPTL3 production. We also demonstrated that the catalytic activity of LPL was elevated upon incubation with extracellular proteins obtained from tangeretin-treated cells. These data indicate that tangeretin reduced the secreted form of ANGPTL3, which may have led to the restoration of LPL activity. Recently, pharmacological inhibition of ANGPTL3 has been found to be effective in the regulation of plasma TG and LDL-C levels. However, the cost-effectiveness of the currently approved ANGPTL3 inhibitor, the neutralizing antibody evinacumab [59], is questionable. To date, the absence of orally administered agents for ANGPTL3 inhibition has limited the therapeutic effect of these novel medications. Our findings suggest that tangeretin, a dietary flavonoid phytochemical, has potential bioactivity for the suppression of ANGPTL3 expression and may serve as a preventive or therapeutic agent for lipid management.

The transcriptional regulation of the *ANGPTL3* promoter has been reported to be primarily controlled by LXRα. LXRα is mainly expressed in the liver and intestine and is a ligand-dependent nuclear receptor that plays an essential role in the regulation of dyslipidemia [60,61]. LXRα can form a heterodimer with retinoid X receptor (RXR) and interact with LXREs in the promoters of target genes. In the presence of ligands, LXRα can bind to ligands, release corepressors, and recruit coactivators to activate the transcription of target genes [62]. The synthetic LXRα ligand T0901317 augmented ANGPTL3 expression in hepatic cells and elevated TG accumulation in the liver and plasma of mice [43,63,64]. In this study, the protein level of LXRα was not changed by tangeretin treatment of hepatic cells; however, T0901317 could dramatically induce ANGPTL3 transcription, which was consistent with previous studies. The LBD of LXRα is located at amino acid residues 206−447 and has been reported to fold into a hydrophobic pocket suitable for small-compound interactions [65]. In this study, molecular docking predicted that tangeretin binds specifically positions in the LBD of LXRα. The docking results showed that hydrophobic amino acids within the ligand-binding pocket of LXR seem to preferentially interact with lipophilic tangeretin via methoxy groups in this PMF. In this study, LXRα ligand-induced ANGPTL3 expression was significantly ameliorated by tangeretin in HepG2 cells. These findings indicate that tangeretin may specifically bind to the hydrophobic pocket of the LBD, resulting in a reduction in LXRα-mediated transcription. Whether tangeretin can directly bind to hepatic LXRα protein remains unclear and requires further determination of direct compound–receptor interaction by ligand binding analysis such as surface plasmon resonance (SPR) assay, circular dichroism spectroscopy, or LC/MS-based analysis in the future [66,67,68]. In addition to LXRα regulation, whether tangeretin can control hepatic ANGPTL3 gene expression via an LXR-independent pathway is unclear, and further clarification is required.

In conclusion, our study demonstrated that tangeretin is a potential ANGPTL3 inhibitor. Tangeretin can modulate the LXRα-ANGPTL3-LPL pathway to regulate lipoprotein metabolism and plasma lipid homeostasis. Our findings reveal that tangeretin has potential efficacy for the prevention and treatment of dyslipidemia and ASCVDs. Here we demonstrated the lipid-modulating effects of tangeretin in cell lines. To support tangeretin as a potentially preventive or therapeutic agent for dyslipidemia by downregulating ANGPTL3 expression, in vivo studies using animal models of dyslipidemia are required.

## 4. Materials and Methods

### 4.1. Chemicals

Tangeretin, dimethyl sulfoxide (DMSO), sodium pyruvate, nonessential amino acids (NEAAs), T0901317, and other chemicals were purchased from Sigma-Aldrich Co. (St. Louis, MO, USA) unless otherwise indicated. DMEM and fetal bovine serum (FBS) were obtained from Thermo Fisher Scientific Inc. (Rockford, IL, USA).

### 4.2. Cell Culture and Compounds Treatment

HepG2 and Huh-7 cells obtained from the Bioresource Collection and Research Center (BCRC, Hsinchu, Taiwan) and American Type Culture Collection (ATCC) were maintained in DMEM containing 10% FBS and 1x NEAA solution in a 5% CO_2_ incubator at 37 °C. Cells were treated with vehicle (0.1% DMSO) and tangeretin for 24 h. For LXRα agonist (T0901317) treatment, the cells were pretreated with tangeretin (40 μM) for 1 h, followed by treatment with T0901317 (1 μM) for an additional 24 h.

### 4.3. Analysis of Cell Viability by MTT Assay

The viability of cells was analyzed by MTT assay as previously described [69]. Briefly, cells were treated with vehicle (0.1% DMSO) or tangeretin (5, 10, 20, 40, and 60 μM) for 24 h, followed by incubation with MTT reagent (1 mg/mL) at 37 °C for 3 h. DMSO was used to dissolve the formazan crystals, and the absorbance was measured at 550 nm.

### 4.4. Analysis of Differentially Expressed Genes (DEGs) by cDNA Microarray

RNA preparation and microarray analysis were carried out as previously described [70]. Briefly, HepG2 cells were treated with vehicle (0.1% DMSO) or tangeretin (40 μM) for 24 h, and total cellular RNA was isolated using TRIzol reagent (Thermo Fisher Scientific) according to the manufacturer’s instructions. RNA concentration and purity were measured and verified for acceptable quality. RNA integrity was analyzed using the Agilent RNA 6000 Nano assay (Agilent Technology, Inc., Santa Clara, CA, USA) and an RNA integrity number (RIN) value >6. Amino allyl antisense RNA (aa-aRNA) was produced by Eberwine-based amplification with an Amino Allyl MessageAmp II aRNA Amplification Kit (Ambion, CA, USA). The aRNAs were labeled with Cy5 fluorescent dye for hybridization with Human Whole Genome One Array Version 7.1 (HOA 7.1, Phalanx Biotech Group, Hsinchu, Taiwan). The fluorescence intensity of each spot was analyzed by GenePix 4.1 (Molecular Devices, Sunnyvale, CA, USA). Genes with expression differences at a *p* value < 0.05 were identified as DEGs.

### 4.5. Pathway Enrichment, Gene Set Enrichment Analysis (GSEA), and Protein–Protein Interaction (PPI) Analysis

A whole set of significant DEGs (*p* < 0.05) in the cDNA microarray was analyzed for Gene Ontology (GO) term enrichment to determine the biological processes (BPs) in which tangeretin-treated cells are involved. The “Reduce + Visualize Gene Ontology” (REVIGO) web-based tool (http://revigo.irb.hr/, accessed on 10 September 2021) was used to summarize and remove redundant GO terms [71]. Gene set enrichment analysis (GSEA) [72] was used to determine the statistically significant BPs in which the tangeretin-treated cells were involved. Free GESA online software was used (http://software.broadinstitute.org/gsea/, accessed on 10 September 2021). The DEGs in the gene set that primarily contribute to the enrichment score (ES) and leading-edge subset may have an essential role in a particular molecular pathway or biological process. The protein–protein interactions (PPIs) were analyzed using the STRING database (version 11) [73] (https://string-db.org/, accessed on 10 September 2021).

### 4.6. RNA Extraction and Reverse Transcription-Quantitative PCR (RT-Q-PCR) Analysis

RNA extraction and RT-Q-PCR analysis were performed as previously described [74]. Briefly, cells were treated with vehicle or tangeretin (20 and 40 μM) for 24 h, and cellular RNA was extracted using a FavorPrep^TM^ blood/cultured cell total RNA purification mini kit (FAVORGEN Biotech, Ping-Tung, Taiwan) according to the manufacturer’s instructions. cDNA was synthesized with a High-Capacity cDNA Reverse Transcription Kit (Thermo Fisher Scientific, Rockford, IL, USA). Quantitative real-time PCR was performed in mixtures containing cDNA, human-specific primers (ANGPTL3, 5′-TCCTGCTGAATGTACCACCA-3′ (forward), and 5′-TCTTCTCTAGGCCCAACCAA -3′ (reverse); GAPDH, 5′-ATGAGAAGTATGACAACAGCCT-3′ (forward), and 5′-AGTCCTTCCACGATACCAAAGT-3′ (reverse)) and Maxima SYBR Green/ROX qPCR Master Mix (Thermo Fisher Scientific, Rockford, IL, USA) reagents. PCR amplification was completed in a Roche LightCycler^®^ 480 Real-Time PCR System (Roche Diagnostics GmbH, Mannheim, Germany) according to the manufacturer’s instructions. The ΔΔCt method was used to calculate the relative differences in mRNA expression. The experimental mRNA levels were normalized to the expression of control GAPDH mRNA in the same samples.

### 4.7. Western Blot Analysis

Cells were treated with vehicle or tangeretin (20 and 40 μM) for 24 h. Total cellular proteins were extracted using RIPA buffer (Thermo Fisher Scientific, Rockford, IL, USA). For nuclear extract preparation, cells were harvested, and nuclear proteins were prepared using NE-PER nuclear and cytoplasmic extraction reagent (Thermo Fisher Scientific, Rockford, IL, USA). The proteins were separated by 10% or 12% SDS–PAGE and transferred onto a PVDF membrane (PerkinElmer, Boston, MA, USA), followed by incubation with specific primary antibodies for human proteins, including anti-ANGPTL3 (ABclonal, Woburn, MA, USA), anti-LXRα (Abcam, Cambridge, MA, USA), anti-HNF-1α (Cell Signaling Technology, Danvers, MA, USA), anti-HDAC2 (GeneTex, Irvine, CA, USA), and anti-actin (Millipore Sigma, St. Louis, MO, USA). The blots were incubated with the appropriate horseradish peroxidase (HRP)-conjugated secondary antibodies (GeneTex, Irvine, CA, USA) at room temperature. The protein signals were detected using Amersham ECL^TM^ prime Western reagents (GE Healthcare, Buckinghamshire, UK), and chemiluminescence-exposed Amersham Hyperfilm^TM^ ECL film (GE Healthcare, Buckinghamshire, UK) was analyzed.

### 4.8. Measurement of Extracellular ANGPTL3 Protein by ELISA

Cells were treated with vehicle or tangeretin (20 and 40 μM) for 24 h. For preparation of secreted proteins, the supernatant of the compound-treated cells was collected and centrifuged to remove the cells. The levels of extracellular ANGPTL3 protein in supernatant samples were measured using a RayBio^®^ Human ANGPTL3 ELISA Kit (RayBiotech, Norcross, GA, USA) according to the manufacturer’s instructions. The absorbance was measured at 450 nm.

### 4.9. Measurement of Lipoprotein Lipase (LPL) Activity

HepG2 cells (1 × 10^6^/mL) were seeded and cultured in DMEM containing 10% FBS and 1x NEAA solution in a 5% CO_2_ incubator at 37 °C for 24 h. The medium was then changed to serum-free medium containing vehicle or tangeretin (40 μM) for an additional 24 h. The extracellular proteins in the culture medium of the compound-treated cells were concentrated with a Vivaspin 20 centrifugal concentrator MWCO 10 kDa (Sigma–Aldrich, St. Louis, MO, USA). LPL activity was measured using a lipoprotein lipase (LPL) activity assay kit (Fluorometric) (Cell Biolabs, San Diego, CA, USA) according to the manufacturer’s instructions. Briefly, the lipoprotein lipase enzyme (15.625 mUnits/mL), the concentrated protein samples (50 μg), and fluorescent substrates were incubated at 37 °C for 90 min on a 96-well plate (black bottom) in a Varioskan Flash spectral scanning multimode reader (Thermo Fisher Scientific, Rockford, IL, USA). The fluorescence intensities were measured under the following conditions: λ_excitation_ = 485 nm, λ_emission_ = 525 nm, repeat: 18 × 5 min.

### 4.10. Construction of ANGPTL3 Promoter–Luciferase Reporter Plasmids

The promoter region of *ANGPTL3* extended from nucleotides −980 to +20 (relative to the A (+1) in the initiation codon ATG) and was prepared by PCR using human genomic DNA (Promega, Madison, WI, USA) as a template. The DNA fragment was amplified and inserted into a pGL3-Basic vector (Promega, Madison, WI, USA), and this construct was called ANGPTL3 p(−980/+20). Plasmids containing 5′ serial deletions, namely, ANGPTL3 p(−750/+20), ANGPTL3 p(−500/+20), ANGPTL3 p(−250/+20), and ANGPTL3 p(−120/+20), were generated using the ANGPTL3 p(−980/+20) plasmid as a template for PCR amplification. The DNA products were inserted into a pGL3-Basic vector. The aforementioned DNA fragments of the *ANGPTL3* promoter region were generated by PCR using specific oligonucleotide primers (Table 2). The DNA fragments produced by PCR were verified by DNA sequencing.

### 4.11. Transfection and Luciferase Activity Assay

Cells were cotransfected with the *ANGPTL3* promoter–luciferase reporter plasmids and the pRL *Renilla* Luciferase Control Reporter Vector (Promega, Madison, WI, USA) using Lipofectamine 2000 Reagent (Thermo Fisher Scientific, Rockford, IL, USA). After transfection for 24 h, the cells were treated with vehicle or tangeretin (20 or 40 μM) for 24 h. The luciferase activity of the transfected cells was determined with a Dual-Luciferase Reporter Assay System Kit (Promega, Madison, WI, USA) and normalized to the activity of *Renilla* luciferase.

### 4.12. Molecular Docking of Tangeretin to the Ligand-Binding Domain (LBD) of the LXRα Protein

Molecular docking analyses were carried out as previously described [45,74]. Briefly, the preferable binding poses of small compounds docked to the LXRα receptor was determined by using Molecular Operating Environment software (MOE2019.01). The “Induced fit” refinement within the DOCK module of MOE software was utilized to enhance the accuracy of predicted binding sites between LXRα and tangeretin or T0901317. Tangeretin and T0901317 were manually built with the MOE software package and docked with the LXRα-binding domain (PDB code: 3IPQ, LXRα with compound GW3965). The crystal water molecules were removed, the missing short loops were added using MOE software, and the energy was minimized before molecular docking. The scoring function used to calculate the binding free energy between ligand and receptor was the force field-based function GBVI/WSA. The preferred binding sites for each compound were determined based on the lowest binding free energy, which is the lowest S value of the scoring function.

### 4.13. Statistical Analysis

All results were confirmed by at least three independent experiments. Each experiment was repeated three times. The data are expressed as the mean ± SD. Statistical analyses were performed using Student’s *t*-test for two-group comparisons. The Levene’s test was performed to determine whether two groups have or have not equal variances before Student’s *t*-test analysis. Comparisons of data with multiple groups were analyzed using one-way ANOVA with Dunnett’s test for post hoc analysis. A *p* value < 0.05 indicated statistical significance.

## Figures and Tables

**Table 1:** The down-regulated genes associated with regulation of lipoprotein lipase activity in tangeretin-treated cells.

Genes	Description	log_2_ (Tangeretin/Vehicle)	*p* Value
*LIPC*	Lipase, hepatic	−1.168	0.0004
*PCSK5*	proprotein convertase subtilisin/kexin type 5	−1.096	0.0001
*FURIN*	furin (paired basic amino acid cleaving enzyme)	−1.063	0.0031
*ANGPTL3*	angiopoietin-like 3	−0.900	0.0479
*ANGPTL4*	angiopoietin-like 4	−0.584	0.0291
*APOA1*	apolipoprotein A-I	−0.558	0.0016
*APOC2*	apolipoprotein C-II	−0.515	0.0080
*APOH*	apolipoprotein H	−0.409	0.0289
*LIMF1*	lipase maturation factor 1	−0.387	0.0268
*HDAC9*	histone deacetylase 9	−0.336	0.0184
*APOC1*	apolipoprotein C-I	−0.323	0.0453

**Table 2:** Primer pairs used in construction of plasmids contained ANGPTL3 promoter DNA region.

Promoter Region	Primer Sequences
p(−980/ +20)	F: 5′-GCGGGTACCTTGCTTGAGCCCAGTATTTC-3′
p(−750/ +20)	F: 5′-GCGGGTACCACGAGCACATGGTAAAGAGC-3′
p(−500/ +20)	F: 5′-GCGGGTACCAGGGAGTGGAGAAAGGCTTC-3′
p(−250/ +20)	F: 5′-GCGGGTACCAAGTTAAATACAATTTCAAA-3′
p(−120/ +20)	F: 5′-GCGGGTACCGTTCACCTACCAACCTTACC-3′
ANGPTL3-Pro-R	R: 5′-GCGAAGCTTAGGAGCTTAATTGTGAACAT-3′

## Data Availability

Not applicable.

## Supplementary Materials

The following are available online at https://www.mdpi.com/article/10.3390/ijms22189853/s1. Table S1: The top 50 of enriched GO terms with the most significant *p* value in tangeretin-treated HepG2 cells.
