# Supplementary material for: The Lipid-Modulating Effect of Tangeretin on the Inhibition of Angiopoietin-like 3 (ANGPTL3) Gene Expression through Regulation of LXRα Activation in Hepatic Cells
Source: Int J Mol Sci. 2021 Sep 12;22(18):9853. doi: 10.3390/ijms22189853 (PMC8471037; doi:10.3390/ijms22189853)
Supplement: Supplementary file 1 [file ijms-22-09853-s001.zip › ijms-1373838-supplementary.pdf]

**Table S1.** The top 50 of enriched GO terms with most significant *p* value in tangeretin-treated HepG2 cells

| ID | Description | *p* value |
|---|---|---|
| GO:0015711 | organic anion transport | 0.000196631 |
| GO:0006820 | anion transport | 0.000372626 |
| GO:0038084 | vascular endothelial growth factor signaling pathway | 0.000460256 |
| GO:0072330 | monocarboxylic acid biosynthetic process | 0.000538243 |
| GO:0033554 | cellular response to stress | 0.000720838 |
| GO:0033044 | regulation of chromosome organization | 0.000722807 |
| GO:0042760 | very long-chain fatty acid catabolic process | 0.001045247 |
| GO:0070482 | response to oxygen levels | 0.001135024 |
| GO:0001666 | response to hypoxia | 0.001172772 |
| GO:0071827 | plasma lipoprotein particle organization | 0.001263218 |
| GO:0043687 | post-translational protein modification | 0.001533743 |
| GO:0045861 | negative regulation of proteolysis | 0.001609589 |
| GO:0043691 | reverse cholesterol transport | 0.001718139 |
| GO:0006996 | organelle organization | 0.001743339 |
| GO:0006629 | lipid metabolic process | 0.001883977 |
| GO:0000725 | recombinational repair | 0.001942245 |
| GO:0006260 | DNA replication | 0.0019534 |
| GO:0002933 | lipid hydroxylation | 0.00201535 |
| GO:0015838 | amino-acid betaine transport | 0.00201535 |
| GO:0046942 | carboxylic acid transport | 0.002027223 |
| GO:0006082 | organic acid metabolic process | 0.002275632 |
| GO:0015910 | peroxisomal long-chain fatty acid import | 0.002333011 |
| GO:0032902 | nerve growth factor production | 0.002333011 |
| GO:0038190 | VEGF-activated neuropilin signaling pathway | 0.002333011 |
| GO:0050722 | regulation of interleukin-1 beta biosynthetic process | 0.002333011 |
| GO:0050725 | positive regulation of interleukin-1 beta biosynthetic process | 0.002333011 |
| GO:1902336 | positive regulation of retinal ganglion cell axon guidance | 0.002333011 |
| GO:0048519 | negative regulation of biological process | 0.002637618 |
| GO:0006950 | response to stress | 0.002680827 |
| GO:0035725 | sodium ion transmembrane transport | 0.002827659 |
| GO:0003333 | amino acid transmembrane transport | 0.002864812 |
| GO:0015849 | organic acid transport | 0.002908723 |
| GO:0032787 | monocarboxylic acid metabolic process | 0.002997776 |
| GO:0006633 | fatty acid biosynthetic process | 0.003089983 |

| | | |
|---|---|---|
| GO:0010876 | lipid localization | 0.003179142 |
| GO:0019752 | carboxylic acid metabolic process | 0.003280088 |
| GO:0035726 | common myeloid progenitor cell proliferation | 0.003400407 |
| GO:0051004 | regulation of lipoprotein lipase activity | 0.003449952 |
| GO:2000573 | positive regulation of DNA biosynthetic process | 0.003486182 |
| GO:0050930 | induction of positive chemotaxis | 0.003674025 |
| GO:0046394 | carboxylic acid biosynthetic process | 0.003766843 |
| GO:0031638 | zymogen activation | 0.004223633 |
| GO:0035924 | cellular response to vascular endothelial growth factor stimulus | 0.004817653 |
| GO:0009611 | response to wounding | 0.004843104 |
| GO:0010951 | negative regulation of endopeptidase activity | 0.004999759 |
| GO:0000724 | double-strand break repair via homologous recombination | 0.005101266 |
| GO:0021800 | cerebral cortex tangential migration | 0.00524607 |
| GO:0071679 | commissural neuron axon guidance | 0.00524607 |
| GO:0048048 | embryonic eye morphogenesis | 0.005272766 |
| GO:0030071 | regulation of mitotic metaphase/anaphase transition | 0.005472417 |
